# Current and Future Therapeutic Approaches of Exocrine Pancreatic Insufficiency in Children with Cystic Fibrosis in the Era of Personalized Medicine

**DOI:** 10.3390/pharmaceutics15010162

**Published:** 2023-01-03

**Authors:** Mirela-Elena Ritivoiu, Cristina Manuela Drăgoi, Dumitru Matei, Iustina Violeta Stan, Alina Crenguţa Nicolae, Mihai Craiu, Ion-Bogdan Dumitrescu, Alina Angelica Ciolpan

**Affiliations:** 1 Faculty of Medicine, “Carol Davila” University of Medicine and Pharmacy, 050474 Bucharest, Romania; 2 Alessandrescu-Rusescu National Institute for Mother and Child Health, 020395 Bucharest, Romania; 3 Faculty of Pharmacy, “Carol Davila” University of Medicine and Pharmacy, 020956 Bucharest, Romania

**Keywords:** pancreatic enzyme replacement therapy, cystic fibrosis, exocrine pancreatic insufficiency, children, CFTR modulators, personalized medicine

## Abstract

This review presents current updates of pancreatic enzyme replacement therapy in children with cystic fibrosis based on literature published in the last decade and some special considerations regarding pancreatic enzyme replacement therapy in the era of new therapies, such as cystic fibrosis transmembrane conductance regulator modulator therapies. Few articles evaluate the efficacy of pancreatic enzyme replacement therapy in the pediatric population, and most studies also included children and adults with cystic fibrosis. Approximately 85% of cystic fibrosis patients have exocrine pancreatic insufficiency and need pancreatic enzyme replacement therapy. Fecal elastase is the most commonly used diagnostic test for exocrine pancreatic insufficiency, although this value can fluctuate over time. While it is used as a diagnostic test, it cannot be used for monitoring the effectiveness of pancreatic enzyme replacement therapy and for adjusting doses. Pancreatic enzyme replacement therapy, the actual treatment for exocrine pancreatic insufficiency, is essential in children with cystic fibrosis to prevent malabsorption and malnutrition and needs to be urgently initiated. This therapy presents many considerations for physicians, patients, and their families, including types and timing of administration, dose monitoring, and therapy failures. Based on clinical trials, pancreatic enzyme replacement therapy is considered effective and well-tolerated in children with cystic fibrosis. An important key point in cystic fibrosis treatment is the recent hypothesis that cystic fibrosis transmembrane conductance regulator modulators could improve pancreatic function, further studies being essential. Pancreatic enzyme replacement therapy is addressed a complication of the disease (exocrine pancreatic insufficiency), while modulators target the defective cystic fibrosis transmembrane conductance regulator protein. Exocrine pancreatic insufficiency in cystic fibrosis remains an active area of research in this era of cystic fibrosis transmembrane conductance regulator modulator therapies. This new therapy could represent an example of personalized medicine in cystic fibrosis patients, with each class of modulators being addressed to patients with specific genetic mutations.

## 1. Introduction

Cystic fibrosis (CF) is one of the most severe and prevalent multisystemic genetic diseases with significant morbidity, especially pulmonary and pancreatic. Chronic lung disease, excessive sweat electrolytes losses, exocrine pancreatic insufficiency, and malnutrition are the most common morbidities.

Although it is predominantly a lung disease displaying chronic inflammation and infection, 85% of patients have exocrine pancreatic insufficiency (EPI), with significant consequences on nutritional status and a substantial impact on survival. According to the European Cystic Fibrosis Society Patient Registry (ECFSPR) 254 patients were registered in Romania in 2020, 94.42% being children. 73% of these patients were diagnosed at the age < 1 year. The average age at diagnosis was 1.47 years [1].

CF is a monogenic, autosomal recessive disorder with highly variable and complex clinical manifestations due to two mutations in the cystic fibrosis transmembrane conductance regulator (CFTR) gene that is situated on the long arm of chromosome 7 and contains 27 exons [2,3].

There are over 2000 mutations with varying functional consequences [4]. The CF gene encodes the CFTR protein. This protein has five domains, two membrane-spanning domains (MSDs), two nucleotide-binding domains (NBDs), and a regulatory domain (R), which acts like a phosphorylation site. On a more detailed view of its role, we find CFTR physiologically expressed on the surface of airway epithelial cells, having a regulatory role considering the chloride ion channel. Consequently to the mutations, the CFTR defective function is reflected upon the impaired transport of chloride (Cl^−^) and bicarbonate (HCO_3_^−^) ions, also limiting the water molecules permeation, encouraging the buildup of mucus at this level, conducting to airway blockage, pathogenic agents accumulation, leading to inflammatory events and infections persistence, irreversibly damaging the lungs, in a relatively short period of time.

Basically, the mucus mobility imprinted by the ciliary domains is affected by the dehydration phenomenon, the bacterial removal and the existence of a sufficient volume of surface liquid are dramatically influenced by the abnormal flow of chloride and bicarbonate ions, and nevertheless, the pH of the fluid is essentially acidic due to the ionic trafficking defect.

The increased viscosity is clinically transposed to airways obstruction, followed by pathogens colonization, triggering infection and inflammation, all resulting shortly in an impaired respiratory process, even severe respiratory failure. This mechanism depicted at the level of the airway epithelial cell develops similarly in other epithelial cells of exocrine glands, e.g., the pancreas [5,6,7].

**Figure 1:**
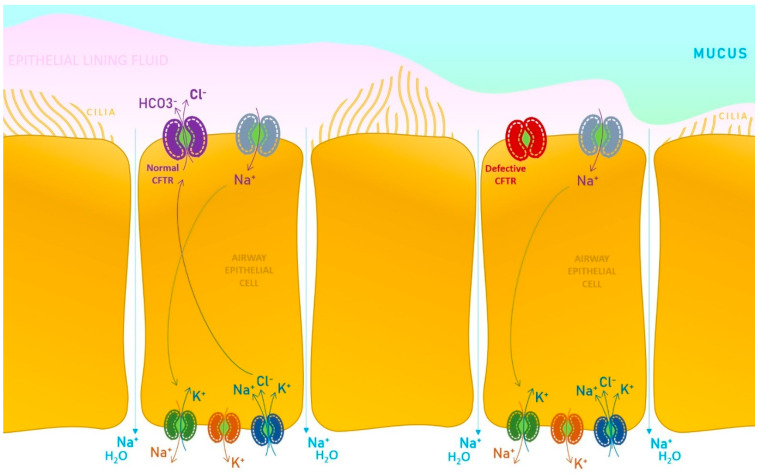
The pathogenic mechanism in cystic fibrosis, at the level of an airway epithelial cell. Briefly, a mutation in the CFTR gene prevents Cl^−^ being excreted from the cell, leading to defective mucous clearance, pathogens clustering and inflammatory events [6,7,8,9].

In a healthy state, the physiologic water content of the airway surface liquid is maintained by a subsequent mechanism facilitated by the absorption of sodium (Na^+^) ions through a dedicated channel (epithelial Na^+^ channel), exiting the cell by the basolateral Na^+^-K^+^ pump. Chloride ions enter the cell by the basolateral Na^+^-K^+^-2Cl^−^ cotransporter and are excreted mainly through the CFTR protein present at the surface of the apical membrane level, which functions as a chloride channel. Secondary, there are also calcium-activated Cl^−^ channels that may partially depurate the chloride from the cell. Considering all the trafficking mechanisms presented, the CFTR physiologic expression and functioning is critical for all other channels that assure a normal electrolytic exchange and a regulatory pathway in the context of secretory tissues (Figure 1).

CFTR protein functions as a chloride and bicarbonate ion channel on the apical membrane of the epithelial cells of exocrine glands. This chloride channel is activated by cyclic adenosine monophosphate (cAMP) [10,11,12].

Defective CFTR results in lower pH of ductal secretion due to reduced bicarbonate buffering, a lower volume, and increased viscosity of digestive and pulmonary secretions. The results are EPI (secondary to duct obstruction with reduced delivery of digestive enzymes to the intestines and impaired absorption of key nutrients) and obstructive lung disease (secondary to abnormally viscous and elastic mucus, that is a hallmark of pulmonary disease, leading to decreased mucociliary clearance, and increased risk for inflammation and infection). CF also determines impairing function of other organs such as the liver, gallbladder and intestines [13,14,15].

Considering that CF is associated with EPI, a common complication of the disease, it suggests that CFTR function has an important role in pancreas physiology [16] and this may be an argument for improving pancreatic function in patients who received CFTR modulators. The effects of these new therapies on pancreatic manifestations are not completely understood yet.

Some recent studies noticed that advances in CF treatment (i.e., CFTR modulators) facilitate a partial restoration of pancreatic function, the underlying mechanism remaining unclear [17]. Pancreatic dysfunction in CF results from ductal obstruction occurring early in life, even in utero [18]. Although the classical theory says that the pancreas is irreversibly damaged in this disease, modulator treatments could have a positive effect on pancreatic function. EPI refers to inadequate production of pancreatic enzymes, bicarbonate, and fluid, which leads to maldigestion and malabsorption.

PERT is the treatment for CF patients that also develop EPI. It is recommended to initiate PERT if there is a strong clinical suspicion of EPI (growth failure, gastrointestinal symptoms, steatorrhea, patients with two CFTR gene mutations associated with EPI) or if EPI is proven by laboratory evidence (fecal elastase less than 200 mcg/g, increased fecal fat, etc.). In general, PERT is administrated at the beginning of the meals, but some clinicians prefer the administration at halfway of the meal. Using PERT after a meal is not as effective.

PERT dose depends on the patient weight or dietary fat intake. For children younger than four years, the starting dose is 1000 lipase units/kg body weight/meal. Children older than 4 years start with 500 lipase units/kg body weight/meal. For infants and tube-feeding children we use the fat-based method. This method recommends the starting dose at approximately 1600 units lipase/g of fat taken per day. The most common causes for PERT failure are inadequate doses and treatment non-compliance (does not respect the timing of doses in relation to meals, the patient forgets to take PERT). If the dose of PERT is more than 10,000 lipase units/kg body weight/day there is a high risk for fibrosing colonopathy. Other side effects are dizziness, abdominal pain, flatulence and perianal rash. Pancreatic enzyme therapy is effective and generally well tolerated by pediatric patients with CF.

Despite significant advances in CF treatment, such as better control of chronic pulmonary infections, accessibility of new therapies, including CFTR modulators, aggressive nutritional supplementation with pancreatic enzymes, and lung transplantation, CF remains a progressive, lethal disease [19].

The current studies on CFTR modulators in the pediatric population are focused on the impact of pulmonary function because CF is predominantly a lung disease with significant pulmonary exacerbations and an important impact on survival. Observational studies on patients who were treated with these new therapies noted different grades of pancreatic recovery.

This review explores current updates on the clinical presentation of CF patients with EPI, the efficacy and safety of PERT, aspects related to doses in children, and special consideration of EPI and PERT in the era of CFTR modulator therapies.

## 2. Exocrine Pancreatic Insufficiency in Children with Cystic Fibrosis

A normal functioning pancreas secrets into the duodenum digestive enzymes and bicarbonate for digestion of macronutrients like protein, fats, and starch. CF is the most common cause of EPI in the children population. 80 to 90% of patients diagnosed with CF have EPI [20,21]. According to ECFSPR—Annual Data Report 2020, the percentage of patients from Romania who were pancreatic insufficient and received pancreatic enzyme was 98% [1].

In EPI, due to CF, pancreatic digestive enzyme secretion is severely decreased, and the amount of bicarbonate is reduced due to obstruction of pancreatic ducts and abnormal secretion. The pancreas of children with CF and EPI is shrunken, with significant fibrosis, cysts, and fatty replacement. Studies showed that pancreatic small duct obstruction starts in utero, in the second trimester of gestation.

The genotype of children with CF is highly predictive of pancreatic function, the grades of EPI being closely correlated with type of mutation. Children with a minimum of one mutation from class V or IV CFTR defects (considered mild mutations) are almost always pancreatic sufficient. By contrast, severe mutations like mutations from class I, II, and III are associated with pancreatic insufficiency [22,23]. In their study, Walkowiak et al., demonstrated that the genotype of a CF patient is strongly linked with the probability of developing exocrine pancreatic insufficiency but not always the presence of a mild mutation excludes pancreatic insufficiency [23]. Insufficient pancreatic patients must immediately initiate standard therapy with pancreatic enzymes.

## 3. Clinical Considerations of Exocrine Pancreatic Insufficiency

### 3.1. Presentation

When the pancreas does not produce physiologic amounts of digestive enzymes results in fats, proteins, and carbohydrates malabsorption. Clinically, patients may manifest malnutrition, poor weight gain or weight loss, and gastrointestinal symptoms such as abdominal distension, steatorrhea, abdominal pain, flatulence, diarrhea, and rectal prolapse. Also, when fats aren’t adequately digested, there is an important risk of constipation or other severe complications like distal intestinal obstruction syndrome.

### 3.2. Diagnosis

There are direct and indirect methods for testing exocrine pancreatic function. In the pediatric population, direct tests are unsuitable because they require endoscopy, an expensive and invasive procedure. However, their high specificity and sensitivity are considered the gold standard for evaluating exocrine pancreatic function. A direct method for assessing the exocrine pancreatic function is performing a secretin-cholecystokinin stimulation test. Direct tests measure the amount of pancreatic enzymes and bicarbonate secreted. Indirect tests are more frequently used than direct tests because they are non-invasive, less expensive, and require less time. They evaluate the effect due to the lack of an enzyme [24,25,26,27].

The most used indirect test is faecal elastase because it is easy to perform, is rapid compared to coefficient of fat absorption (CFA) or faecal chymotrypsin, evaluates the need for PERT, and is more specific and sensitive compared with faecal chymotrypsin and lipase [27,28,29]. Unlike faecal chymotrypsin and faecal lipase, stool elastase does not require discontinuation of PERT before dosing, does not require special storage, and one stool sample is enough for the assessment. Patients with CF and EPI usually have levels < 15 mcg/g stool of faecal elastase.

Healthy people have faecal elastase >200 mcg/g stool (usually >500). Severe pancreatic insufficiency is diagnosed when faecal elastase <100 mcg/g stool, and mild/moderate pancreatic insufficiency is between 100–200 mcg/g stool [30]. Patients who are pancreatic sufficient at diagnosis need periodic assessment due to the risk of becoming pancreatic insufficient by age.

### 3.3. Malnutrition in CF

Undernutrition is strongly associated with CF and is correlated with genetic factors (i.e., mutations) and with other factors, such as higher energy needs (120–150% of normal requirements), energy losses, and decreased nutrient intake and absorption. CF patients have an increased basal energy expenditure resulting from chronic pulmonary infections and breathing efforts. Although improved nutritional status was observed in the last 2 decades, adequate nutrition remains an important issue. Children with CF associate a poor nutritional status that leads to declined lung function and increased mortality.

Patients that were early diagnosed through newborn screening programs received an early nutritional therapeutic intervention, and thus, they could have a positive nutritional outcome [31]. Newborn screening for CF became widely available in the last few years. According to European Cystic Fibrosis Society Patient Registry (ECFSPR), Annual Data Report 2020, 79% of children of 5 years old or younger, who were registered in 2020, were screened at birth. However, Romania has no neonatal screening program for CF yet, consequently, only 9% of patients registered in 2020 were screened at birth [1]. In the absence of this program, for an early diagnosis, the best screening procedure is a careful monitoring of growth, a detailed medical history, and a physical exam.

Three conditions contribute to malnutrition in CF patients: energy losses, high-energy needs, and inadequate nutrient intake. The essential cause of energy loss is represented by maldigestion/malabsorption due to EPI. Energy needs are higher in CF patients with pancreatic insufficiency than in healthy individuals. High-energy expenditure seems to be correlated with pancreatic insufficiency. High-energy requirements are associated with lung inflammation and infection. Basic nutritional care includes a high-calorie, high-fat diet associated with PERT, and fat-soluble vitamins supplementation. Morbidities like pulmonary infections, gastrointestinal complication (gastro-esophageal reflux, constipation, distal intestinal obstructive syndrome) can decrease appetite and determine inadequate nutrient intake [31,32,33].

Other complications of CF are represented by a CF-related diabetes, that could determine and worsen malnutrition and CF-related liver disease which associates specific nutritional deficiencies (fat-soluble vitamins, essential fatty acid, calcium), that can lead to osteopenia and osteoporosis [34,35,36,37].

Considering the consequences of nutritional deficiencies in children with CF it is recommended an early and sustained nutritional therapy, to obtain adequate growth, similar to that of same-aged non-CF patients. Patients with body mass index (BMI) values below 25th percentile have been considered nutritionally “at risk”. According to the European Society for Clinical Nutrition and Metabolism (ESPEN) the European Society for Paediatric Gastroenterology, Hepatology and Nutrition (ESPGHAN) and the European Cystic Fibrosis Society (ECFS) guidelines on nutrition care for children below 2 years of age are recommended both weight and length for age percentiles to assess the nutritional status, and the aim is achieving the 50th percentile. For children above 2 years, and adolescents (2–18 years) are recommended regular assessment of weight, length and BMI, and the goal is achieving 50th percentile for healthy children [38]. In Romania the median z-score for BMI for children 2 to 17 years registered in 2020 was −1 (meaning that 50% of the patients are below of this z-score for BMI). The percentage of children underweight (z-score < −2) for patients 2 to 17 years was 20% for the female sex, and 18% for male patients [1]. BMI is used to evaluate nutritional status, but it has limitations because it could not differentiate fat mass from lean body mass (LBM). Another 2 indicators of nutritional deficiency are more sensitive than BMI: LBM and bone mineral content (BMC), lower values being associated with impaired pulmonary function in children [39,40]. BMI fails to assess body composition, especially fat free mass, an important indicator of nutritional status. Lower values for fat free mass were associated with decreased pulmonary function and undernutrition. Also, pulmonary chronic infection, a characteristic of this disease has been linked to decreased fat free mass [41,42]. The predictive value of body composition for clinical outcomes of CF patients is still undefined [43]. The prevalence of overweight and obesity is increasing in the last years, among adult patients with CF [44,45].

Research suggests that CFTR modulators may increase weight in CF patients, the underlying mechanism being multifactorial (improved pancreatic exocrine function, increased calorie intake, and nutrient absorption, decreased energy expenditure due to improved pulmonary function) [34,46,47].

According to Cystic Fibrosis Foundation (CFF) Patient Registry—annual data report 2020, 40.4% of adult patients are overweight (28.7%) or obese (11.7%), with a high prevalence in men. This percentage has more than doubled in the past 20 years (15.3% in 2001) [48].

## 4. Treatment

Nutritional status has a significant role in patients’ outcomes. Early diagnosis, better nutritional and PERT management, improved antibiotics regimen and pulmonary clearance therapy, and use of new therapies—CFTR modulators, determined an improved outcome of CF patients in the last decades [49,50].

It is recommended to introduce PERT once pancreatic insufficiency is clinically manifest. These products contain pancrelipase extracted from swine pancreas. Pancrelipase is a mixture of enzymes that provides lipase, amylase, and protease that ensures the digestion of fats, proteins, and starch in order to improve growth, weight gain, and developmental processes. The first pancreatic enzyme supplements were in powder form and non-enteric tablet form; further studies have shown that they were only partially effective on symptoms of EPI.

Pancreatic supplements usually act in the alkaline environment of the duodenum. In the case of the acidic pH of the gastric milieu, they are inactivated, and this was the reason for changing the previous formulations to enteric-coated preparations and enteric-coated microspheres, which significantly increased their efficiency.

CF and EPI patients require long-term therapy. The current recommendation for PERT is to be taken before and/or during a meal but not after it because it is ineffective [51,52]. It is recommended that pancreatic enzyme supplements be administered to all children with CF and EPI as soon as possible, with CF-fed infant formula or solid food [51,52,53]. Also, the capsule content could be mixed with applesauce, a soft non-alkaline food, infant formula, or fruit puree and administered right before the meal.

It is recommended that the capsules be distributed over the entire day based on the fat content of the foods. The coefficient of fat absorption has improved when PERT has been taken considering the fat content of the foods consumed.

There is insufficient information in the literature about the adequate dose of lipase that should be taken. Excessive doses of pancreatic enzymes may cause abdominal pain or constipation [54,55,56]. There are two ways to administrate PERT: by weight and based on the amount of fat intake. By weight, it is recommended to begin with 1000 lipase units/kg/meal if the child is less than four years old. Children over this age start with 500 lipase units/kg/meal and never more than 10,000 lipase units/kg body weight/day because of the fibrosing colonopathy risk [52,57,58]. The fat-based method is more valuable in the case of patients with a feeding tube, or when the amount of fat intake can be measured. Usually, it begins with 2000 lipase units/120 mL of formula or breast milk [59]. Table 1 presents a synthetic description of PERT in children, the available pharmaceutical products, their lipase concentration and posology.

Nowadays, in clinical practice it is very difficult to monitor the efficacy of PERT and determine the need for dose adjustment. The absence of a method for PERT dosage adjustments constitutes an essential aspect of clinical practice. Usually, PERT adjustment is guided by gastrointestinal symptoms like abdominal pain, diarrhea, greasy stools, and bloating [61,62]. When a patient does not have an optimal response with an almost maximal dosage of PERT, the medical practitioner should consider reducing gastric acidity by adding histamine 2 receptor antagonists, as famotidine, or proton pump inhibitors (omeprazole) [63]. Faecal elastase is not an adequate method for monitoring the effectiveness of PERT and dose adjustments. In research papers, for evaluating the efficacy of PERT on EPI due to CF, the coefficient of fat absorption (CFA) has been the most commonly used primary endpoint. CFA is calculated from fat intake (100 g/day of dietary fat) and excretion (from 72 h of faecal collection) using the formula:

CFA (%) = [(fat intake in g-fat excretion in g)]/fat intake in g × 100

CFA measures the effect of PERT on fat absorption in EPI. The coefficient of nitrogen absorption (CNA) measures the effect of PERT on protein absorption in EPI [64,65,66].

Some studies reported fibrosing colonopathy associated with high doses of PERT [67] In contrast, other studies described a lower efficacy of some types of PERT [68], but overall, based on well-controlled clinical studies and clinical experience, PERT is considered well-tolerated and effective [66,69,70,71]. As a marker of fat malabsorption, CFA was improved in over 85% of patients who had received PERT [72].

In a double-blind, placebo-controlled study, Stern et al., showed that PERT improves faecal fat absorption and decreases gastrointestinal symptoms [66]. A challenge in CF patients’ management is represented by the complex and time-consuming treatments that lead to low adherence to medications. Adherence to PERT plays an important role in nutritional status and in reducing the risk of hospitalization in CF children patients. A study conducted by Trapnell et al., demonstrated a correlation between adherence to PERT and increased body mass index (BMI) with a decrease in hospitalization rate, strengthening the observation that there is a close association between nutritional status and clinical outcomes. It is well known that in children with CF, reduced lung function is associated with an increased rate of hospitalization, and poor nutritional status, whereas a better nutritional status has a positive impact on lung function and survival rate [38,73,74].

Improving adherence to PERT is a key challenge for the prevention of malnutrition, and thus for increasing quality of life, and for improving pulmonary disease’s outcome [75].

Nutritional care should be an important key point of management of CF. The aim is represented by obtaining a normal growth pattern in children with CF. Increasing caloric and supplements intake, optimizing PERT and pulmonary management, in order to obtain an adequate nutritional status, still remain an important goal of treatment [76]. Very few studies have examined the risk factors for malnutrition among pediatric population with CF, and most of them refers to BMI status and impairment pulmonary function [77].

In 2016 ESPEN, ESPGHAN and ECFS published a guideline with nutritional recommendation for CF patients. The recommendations are focused on adjusting energy intake upward to achieve a normal growth, balancing protein and fat intake. The energy target for children is 110–200% of energy requirements for the same age healthy children. Pancreatic insufficiency can lead to fat-soluble vitamin deficiency, particularly vitamins A, E and K, but also vitamin D. 10–35% of pancreatic insufficient patients have fat-soluble vitamin deficiencies and need vitamin supplementation [31,78].

A study conducted by Ashkenazi et al., has been demonstrate that malnutrition in children with CF was associated with an increased risk of malnutrition in adulthood, and also represented a risk factor for lung transplantation [79].

Another study (Smith et al.) reveals a significant lower mortality rate for pediatric CF patients admitted in Pediatric Intensive Care Unit (PICU). Risk factors for increased mortality rate were represented by malnutrition, along with renal and cardiovascular comorbidities, specific pulmonary complications, such as hemoptysis/pulmonary hemorrhage, pneumotorax and chronic airway obstruction, respiratory intervention particularly invasive ventilation and gastrointestinal complications (hepatobiliary disease, gastrointestinal bleeding, splenomegaly) [49].

## 5. PERT and CFTR Modulator Therapies

CF is a genetic disease caused by various CFTR gene mutations that lead to the dysfunction of CFTR. Establishing a prognosis in CF patients, especially in children is a huge challenge considering that it is extremely difficult, due to important genotype-phenotype variability. Patients with the same mutation (e.g., F508del) may present different clinical outcomes, from severe to mild courses [80].

Regarding pancreatic function, CFTR is essential for ductal fluid production, bicarbonate secretion, and adequate production of pancreatic enzymes. The reduction in pancreatic function can be variable and correlated with the severity of CFTR mutation. Approximately 85% of CF patients are pancreatic insufficient and have maldigestion requiring therapeutic intervention [81,82,83].

Acute pancreatitis is a rare complication of CF in the pediatric population and usually occurs in pancreatic-sufficient patients [84].

The CFTR glycoprotein is mostly expressed at the apical membrane level of epithelial cells within a variety of exocrine tissues, being involved mainly in fluid homeostasis. It has several functional subunits, reunited in the form of two membrane-spanning domains (MSD) that factually constitute the channel pore which opens and closes upon conformational changes, a regulatory (R) domain acting as a phosphorylation site driven by cAMP-dependent protein kinase A and C, and two nucleotide-binding domains (NBD), whose association with adenosine triphosphate (ATP) initiates conformational changes and their dimerization in a head-to-tail configuration, specific to the open state of the channel [85].

The CFTR protein maturation is a complex process of folding, assembling, and glycosylation, which is performed primarily in the endoplasmic reticulum upon translation and is accomplished later on in the Golgi apparatus. This intricate process can be subjected to a series of mismatching events or disruptions in various steps, conducting to a high rate of degradation by ubiquitination or autophagy. The mature functional CFTR protein finally reaches the plasmatic membrane, where it displays a high degree of stability and undergoes the internalization procedure.

**Figure 2:**
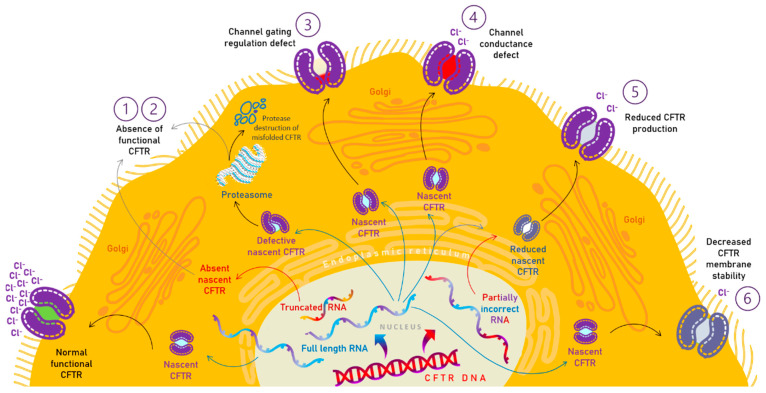
The six mutation classes of cystic fibrosis transmembrane conductance regulator (CFTR) gene. The defective mechanisms (1–6) are presented in comparison with the physiologic process encountered in healthy subjects. [6,7,8].

CFTR mutations are depicted by six different classes, considering the particular defect in protein synthesis, trafficking, function, or stability, although many CFTR mutants present multiple defects [86] (Figure 2).

Class I mutations, implying a protein synthesis defect, present severely defective protein production. Due to stop codons or splicing defects, no functional CFTR protein is synthesized. In this class are included G542X, W1282X, and R553X mutations. According to the CFTR2 database [4], G542X causes pancreatic insufficiency in 98% of the patients, W1282X in 99% of them and R553X in 97%. As reported by the European Cystic Fibrosis Society Patient Registry (ECFSPR)—Annual Data Report 2020 G542x was the most second common mutation in Romania, after F508del [1].

In class II mutations (maturation defect), CFTR protein is synthesized but abnormally folded so it is degraded intracellularly. Impairment of Cl^−^ channel gating is associated and leads to CFTR instability on the apical membrane. F508del is the most common mutation in CF patients. It belongs to class II mutations alongside the N1303K mutation. Both of them are associated with a 98% risk of pancreatic insufficiency. According to ECFSPR, 80.7% of CF patients registered in 2020 have at least one F508del allele. Related to patients registered in 2020, 99.12% have done DNA testing. The prevalence of F508del homozygous was 45% in Romania, and almost 40% for F508del heterozygous, patients [1].

In class III mutation, representing a gating defect, CFTR protein is produced and correctly localized, but the activation and regulation by cAMP are disrupted, so the channel functionality is severely affected. G551D, G178R, S549N, S549R, G551S, G970R, G1244E, S1251N, S1255P, and G1349D are some of the mutations included in this class. Patients with the G970R mutation associate pancreatic insufficiency in 100% of cases, G551D mutation has a 96% risk of pancreatic insufficiency, while those with G551S mutation have only a 33% risk.

Class IV mutations, implying a conductance defect, present channel dysfunction due to reduced chloride conductance, but the CFTR channel is able to open and close. Mutations described in this class generally have a lower risk of pancreatic insufficiency, beginning with G314E which has 0% association with pancreatic insufficiency, R334W mutation has 40%, and the most significant risk has the R347P mutation, which is present in almost 68% of the cases.

In class V mutations, the CFTR protein synthesis is normal but in smaller quantities than usual. Generally, this class was associated with pancreatic sufficiency 2789 + 5G → A mutation has a higher association with pancreatic dysfunction in 43% of the cases, 3849 + 10 kb C → T mutation in 33% of CF cases, while 3272-26 A → G mutation causes pancreatic insufficiency in 29% of patients.

Class VI mutations result in a decreased CFTR stability at the level of the apical membrane due to higher endocytosis rates or lower plasmatic membrane recycling ones [4,6,8,87].

Table 2 reunites data considering the classes of CFTR mutations, the respective percent of risk in developing EPI and therapeutic approaches available, for the consequent classes of CFTR modulators correlated to each type of mutation.

A correlation between genotype-phenotype of pancreatic disease was described in CF patients. Genotype classes I, II, and III are associated with EPI due to a more significant CFTR deficiency or dysfunction. Genotype classes IV, V, and VI or heterozygous individuals with one less severe allele tend to conserve a level of pancreatic exocrine function. Response to treatment varies between different genotype classes and within each class itself [87,88].

CF patient outcomes are also influenced by epigenetic factors, genetic modifiers, environmental factors, and socioeconomic status. CFTR modulators are classified into five groups based on their mechanism: potentiators, correctors, stabilizers, read-through agents, and amplifiers [89].

The potentiator modulators help to maintain proper conductance at the CFTR channel by keeping the gate open for a longer period. The CFTR corrector modulators help CFTR protein return to a 3-dimensional shape so it can move to the cell surface and stay there for a longer time. The stabilizers ensure a better stability at the plasma membrane of the CFTR channel. The read-through agents help continue the translation process despite the encounter of a stop codon ensuring a complete CFTR protein. The amplifier modulators are helpful when CFTR protein is deficient by increasing the amount of CFTR protein in the plasma membrane [87,90,91]. This new therapy targets the underlying CFTR defect and improves its function. Modulator treatments must be individualized and have been used not in all patients but only in CF patients with specific genetic mutations. For every discovered and approved modulator, there is a clear indication based on specific mutations [91]. Four CFTR modulators are used: ivacaftor, lumacaftor-ivacaftor, tezacaftor-ivacaftor, elexacaftor-tezacaftor-ivacaftor.

A combination of two modulator drugs, a corrector and a potentiator, is required to restore the CFTR function of F508del, the most common CFTR mutation [92].

In 2020, the Food and Drug Administration approved the use of a combination of 3 modulators, 2 correctors (tezacaftor and elexacaftor), and one potentiator (ivacaftor) for children 12 years or older with at least one F508del mutation, and since 2021 for children ages 6 through 11 with certain mutations [91].

As a result of the increased use of CFTR modulators, there are described three different patients groups with varying grades of pancreatic insufficiency and different response to therapy. There are insufficient pancreatic patients that may recover their pancreatic function. Another subset of patients treated with CFTR modulators are those with a recovered pancreatic function and who may develop pancreatitis (this fact suggests that treatment could increase pancreatic acinar reserve). The third subset is represented by sufficient pancreatic patients (those who had recurrent episodes of pancreatitis or have a pre-existing risk of pancreatitis) who may have a reduced risk of symptomatic pancreatitis. The risk-benefit ratio of these therapies varies for the 3 subsets of patients, depending on the grade of pancreatic insufficiency and preexisting risk of pancreatitis. Pancreatic dysfunction in CF patients begins early in life. Some studies noticed that CFTR modulators improve and recover exocrine pancreatic function (measured by the level of pancreatic enzymes such as fecal elastase, and immunoreactive trypsinogen), this improvement positively impacting the growth and nutritional status later in life [87,93]. PERT represents the treatment of EPI and is a substitution one, with pancreatic enzymes. It is addressed to a complication of the disease, while CFTR modulators target the defective CFTR protein. These new therapies may determine a partial recovery of pancreatic function and could require adjustment of pancreatic enzyme doses. Instead of this improvement of pancreatic function, CFTR modulators do not represent an alternative treatment for EPI.

Ivacaftor is a CFTR potentiator that increases CFTR protein function on the epithelial cell surface in patients with specific mutation. It has been in clinical use for pediatric patients 6 years old or above for almost a decade. Ivacaftor was the first molecule of this new generation of precision medicine drugs in CF and currently is approved for use in patients aged 4 months and older. Initially was approved for use in CF patients with the G551D CFTR mutation; 4 to 5% of all CF patients have this mutation. It potentiates the CFTR function in patients carrying this mutation [94,95,96]. Nowadays it is approved for 97 mutations [97]. Several studies showed that ivacaftor slows CF disease progression by improving lung function, respiratory symptoms and nutritional status. In their study, Volkova et al., noticed that ivacaftor treated patients for up to 5 years had a lower risk of pulmonary exacerbation [98,99].

Currently, modulator therapy is approved for use in children aged 2 years or older, and, very recently (September 2022), even for children as young as 1-year-old for combination lumacaftor-ivacaftor. Open-label studies showed that treatment with lumacaftor-ivacaftor was generally safe and well-tolerated in children aged 6–11 years with CF homozygous for the F508del-CFTR mutation and also in children aged 2–5 years old [100,101]. In the first personalized monitoring study of different CFTR modulator efficacy in adult patients, Niedermayr et al., showed that there are differences in functioning of CFTR before and after starting treatment with CFTR modulators. Patients with the same mutation (F508del), sex, age, and disease severity have different clinical outcomes, that varies from nearly complete response (for lumacaftor-ivacaftor) to non-responding (for lumacaftor-ivacaftor or tezacaftor-ivacaftor) [80].

The most frecquent mutation, F508del, determines multiple CFTR protein defects that could be controlled by a triple combination of CFTR modulators elexacaftor, tezacaftor and ivacaftor. This association of three modulators will improve CFTR channel trafficking and gatting. The aim of modulators’ association was to rescue CFTR mutants protein presenting more than one type of protein defects [102,103]. This triple therapy (elexacaftor, tezacaftor and ivacaftor) was approved for use in patients aged 6 years and above who have at least one F508del mutation, and is used in combination with ivacaftor This association has demonstrated important positive outcomes in clinical trials [104,105]. Also, it has been demonstrated that this triple combination of CFTR modulators was associated with important increase in weight and BMI, while impact of body composition outcomes needs further investigation [44,106,107,108]. Sporadic cases of non-responders among patients have been reported, probably due to a particular combination of genetic factors, such as genetic variants in enzymes involved in CFTR modulators metabolism (i.e., Cytochrome P450 enzyme), or limited compliance of the patients [103].

The ARRIVAL trial revealed improvement of pancreatic markers (increase of faecal elastase, decrease of immunoreactive trypsinogen and reduction in lipase, and amylase) in children between 12 and 24 months who received ivacaftor, changes that suggest an improvement of pancreatic function [109]. Other studies KIWI/KLIMB demonstrated similar improvements in children aged 2–5 with an increase of faecal elastase, weight, and body mass index z scores. A recent study in ferret models who received ivacaftor in utero reveals partial protection from disease progression. Postnatal administration of ivacaftor improved pancreatic exocrine function and also growth and survival rate [110]. Even if CFTR modulators could lead to improve nutritional status, there is no consistent evidence of their effect on fat absorption. In their study, Mennella et al., reported an improvement in fat absorption in patients treated with ivacaftor [111], but there are no data in CF patients with non-gatting mutations [112].

Thus, these new therapies with modulators improved pancreatic exocrine function even if pancreatic insufficiency in CF patients has been considered irreversible until now. Patients with at least partial restoration of pancreatic function after modulators therapy can develop symptomatic pancreatitis, even though this disease was considered uncommon in CF patients with EPI. Gouda et al., and Megala et al., described patients treated with modulator therapy who developed acute pancreatitis [113,114]. Patients who are pancreatic insufficient rarely develop acute pancreatitis because of their decreased acinar reserve. It suggests clinicians should be alert to pancreatitis symptoms in CF patients treated with these new therapies. Patients with recurrent pancreatitis (pancreatic sufficient patients) treated with CFTR modulators, may have a reduced risk of symptomatic pancreatitis [115].

Pancreatitis must also be considered in the differential diagnosis for pancreatic sufficient and pancreatic insufficient CF patients on CFTR modulator therapies.

Based on significant variability in faecal elastase response, both KIWI and ARRIVAL trials suggest that CFTR modulator therapy can improve and partially restore exocrine pancreatic function in some patients, possibly resulting in the need to adjust enzymes doses. Given this significant variability in response to treatment, approaches to PERT need to be reviewed, aiming to create guidelines for monitoring EPI and the efficacy of PERT and determining a need for dose adjustment.

Modulator therapies represent a fundamental change in CF’s treatment, meaning a transition from addressing complications of a disease resulting from CFTR proteins’ defect, to the restoration of partial function of this defective protein, consequently determining disease modification [116,117].

These new therapies, CFTR modulators, became a cornerstone in CF management. Many studies concluded that these new therapeutic strategies have improved the nutritional status, patients’ quality of life, and even survival rate [118,119,120].

Further studies are necessary for understanding the mechanism involved in improving pancreatic function and also to determine the long-term effects of these new therapies, focusing on life expectancy. Being the most studied CFTR modulator, ivacaftor was analyzed for a period of 5 years. The lower risk of death noted in the first year was similar after 5 years of treatment [121]. Mortality rate decreased over the last 3 decades. As reported by ECFSPR, 19.2% of deaths among CF patients in 2020 were in the age group of 0–20 years, thus deaths in small children (under 5 years) were very rare (4.46%) the most frequent cause being respiratory disease [1].

Still, not all CF patients can benefit from these new therapies, because these modulators are only effective in patients with specific mutation classes since different mutations cause different defects in CFTR protein. An important goal in the field of the CF research is to be able to provide CFTR based therapy to all CF patients. It refers to highly effective CFTR modulators and also to nucleotide and cell-based therapies (mRNA correction or replacement, gene transfer, gene editing, stem cell replacement) [122,123]. CFTR modulators herald a new therapeutic era, a personalized one, in which CF treatment is guided by the patients’ genetic information.

## 6. Conclusions

Most patients with CF have exocrine pancreatic insufficiency that leads to fat malabsorption and malnutrition, and secondary to fat-soluble vitamin deficiences [124]. In CF patients, malnutrition is linked to more severe lung disease and a shorter life expectancy. PERT is a therapy that improves nutritional status and growth and contributes to improved pulmonary function and survival rate.

There is no well-defined standard dose for PERT, and doses should be individualized. The international guidelines aim to keep lipase dose below 10,000 units/kg/day and correlate it with the meal’s fat content. Doses will be adjusted based on gastrointestinal symptoms, weight gain, and growth. Fibrosing colonopathy is a complication of PERT associated with high amounts of lipase. Otherwise, PERT is considered effective and well-tolerated in children with CF.

Recent research in exocrine pancreatic function in this new era of personalized medicine i.e., CFTR modulator therapies have questioned the hypothesis that EPI in CF pediatric population is irreversible. Increasing the use of CFTR modulators will allow us to advance in understanding pancreatic pathophysiology, especially in exocrine pancreatic function.

We consider that further clinical studies are needed to help us to understand the role of CFTR modulators and timing of treatment initiation in preventing EPI and malnutrition in children with CF and thus in improving the quality of life and possible the survival rate.

## Figures and Tables

**Table 1:** Pancreatic Enzymes Replacement Therapy (PERT) in children: Marketed pharmaceutical products, the lipase units’ contents and dose recommendations [27,60].

PERT Pharmaceutical Product	Units of Lipase Contents	Enteric-Coated or Non-Enteric Coated Formulations	Posology/Dose and Administration Recommendations
Kreon/Creon^®^	3000; 6000; 10,000; 12,000; 24,000; 28,000; 36,000	Enteric-coated microspheres	500–2500 U/kg body weight/meal 500–4000 U/g of fat/day 2000–5000 U/breastfeed or 100–120 mL infant formula (Not to be mixed directly with the formula or breast milk) Not to exceed 10,000 U/kg/day
Pankreal^®^	35,000	Non-enteric-coated capsules	
Zenpep^®^	3000; 5000; 10,000; 15,000; 20,000; 25,000; 40,000	Enteric-coated beads	
Pancreaze/Pancrease^®^	2600; 4200; 10,500; 16,800; 21,000; 37,000	Enteric-coated microtablets	
Ultresa^®^	4000; 13,800; 20,700; 23,000	Enteric-coated beads	
Viokase/Viokace^®^	10,440; 20,880 (requires acid suppression: proton pump inhibitor) 8000; 16,000; 16,800 per each 0.7 g powder (¼ teaspoonful)	Non-enteric-coated tablets or powder	
Pertzye^®^	4000; 8000; 16,000; 24,000	Enteric-coated microspheres	
Nutrizym 22^®^	22,000	Enteric-coated tablets	
Pancrex V^®^	2950; 8000	Non-enteric-coated hard capsules	Pancrex V Powder may be administered via a nasogastric or a gastrostomy tube.
	5600 25,000/1 g powder	Enteric-coated tablets or powder	

**Table 2:** Classes of CFTR mutations, risk of EPI and therapeutic approach (adapted from CFTR2—Clinical and Functional Translational of CFTR and McKay et al.) [4,87].

Class	Type of Defect	Risk of EPI	Examples of CFTR Mutations	Therapeutic Approach	Available Drugs
I	protein synthesis defect	98% 99% 97%	G542x W1282x R553x	Genetic therapies Read-through agents	—
II	protein trafficking	98% 98%	F508del N1303K	Corrector (and potentiator)	Elexacaftor/tezacaftor/ivacaftor Lumacaftor/ivacaftor Tezacaftor/ivacaftor
III	gatting defect	100% 96% 33%	G970R G551D G551S	Potentiator	Ivacaftor
IV	conductance defect	0% 40% 68%	G314E R334W R347P	Potentiator	Ivacaftor
V	reduced protein synthesis	43% 33% 29%	2789 + 5G → A 3849 + 10KbC → T 3272-26A → G	Amplifier	—
VI	decreased stability	—	c.120del123 rPhe580del	Stabiliser	—

## Data Availability

Not applicable.
